# Surgical treatment of scoliosis in Treacher Collins syndrome: a case report

**DOI:** 10.1186/1752-1947-8-446

**Published:** 2014-12-19

**Authors:** Christos Karampalis, Nikolaos Bounakis, Athanasios I Tsirikos

**Affiliations:** Scottish National Spine Deformity Centre, Royal Hospital for Sick Children, Sciennes Road, Edinburgh, EH9 1LF UK

**Keywords:** Posterior spinal fusion, Scoliosis, Surgical treatment, Treacher Collins syndrome

## Abstract

**Introduction:**

Treacher Collins syndrome is an autosomal dominant disorder resulting in congenital craniofacial deformities. Scoliosis has not been previously reported as one of the extracranial manifestations of this syndromic condition.

**Case presentation:**

We present a 15-year-old British Caucasian girl with Treacher Collins syndrome who developed a severe double thoracic scoliosis measuring 102° and 63° respectively. The deformity was noted at age 14 years by the local general practitioner and gradually progressed until she was referred to our service and subsequently was scheduled for surgical correction. There were no congenital vertebral anomalies. As part of the condition, she had bilateral conductive hearing impairment. She also had reduced respiratory reserves and a restrictive lung disease. Both curves were rigid on supine maximum traction radiographs. She underwent a single-stage anterior and posterior spinal arthrodesis with pedicle hook/sublaminar wire/screw and rod instrumentation and autologous rib graft, supplemented by allograft bone and made a good postoperative recovery. Her scoliosis was corrected to 25° and 24° and a balanced spine in the coronal and sagittal planes was achieved. At latest follow-up beyond skeletal maturity (3 years post-surgery) she had an excellent cosmetic outcome with no loss of deformity correction, no detected pseudarthrosis and a normal level of activities.

**Conclusions:**

Scoliosis can occur in patients with Treacher Collins syndrome with the deformity demonstrating significant deterioration around the adolescent growth spurt. A high index of awareness will allow for an early diagnosis and scoliosis correction at a stage when this can be safer and performed through a single-stage posterior procedure. If the deformity is detected at a later age and stage of growth as occurred in our patient, more complex surgery is required and this increases the risk for major morbidity and potential mortality. Surgical treatment can correct the deformity, balance the spine and restore cosmesis, as well as prevent mechanical back pain and respiratory complications if the scoliosis progressed to cause severe thoracic distortion. A thorough preoperative assessment can diagnose associated comorbidities and reduce the risk for postoperative complications.

## Introduction

Treacher Collins syndrome (TCS) is an autosomal dominant disorder resulting in congenital craniofacial malformation. Patients have no associated developmental delay or neurological disease. Spinal deformity has not been described as one of the features of this syndromic condition. We present a patient with TCS who developed a severe double thoracic scoliosis and underwent a combined single-stage anterior/posterior spinal arthrodesis. We describe the patient’s postoperative course and final outcome at skeletal maturity, 3 years following scoliosis surgery. To the best of our knowledge, this is the first report of a patient with this condition developing scoliosis and requiring surgical treatment.

## Case presentation

A British Caucasian girl aged 15 years presented to our institution with a double thoracic scoliosis. She was diagnosed with TCS type 1 on the basis of clinical findings and genetic testing. As part of the underlying condition she had bilateral conductive hearing impairment treated with hearing aids. She also had a mild degree of micrognathia with good mouth opening and lower jaw protrusion. There was no history of chest or upper airway infections and she had an effective cough. There was no family history of syndromic conditions or scoliosis.

The development of a scoliosis was first noted at the age of 14 years. No treatment was given at that stage and the deformity gradually progressed. At presentation to our clinic, she was post-menarche with height 162.7cm, arm span 171cm, body weight 50.6kg, and body mass index 31.2.

On clinical examination, she had a severe right thoracic scoliosis which was rotated to the right and was producing a marked prominence of the rib cage and scapula adjacent to the convexity of the curve. There was also thoracic translocation and listing of her trunk to the right with associated waistline asymmetry and prominence of the left side of her pelvis. A left upper thoracic scoliosis was present and this resulted in levelling of her shoulders. Her pelvis was level with no evidence of leg-length discrepancy. There were no skin or soft tissue abnormalities overlying her spine. She reported no neurological abnormality. A neurological examination confirmed normal tone, muscle power, sensation and tendon reflexes in her upper and lower limbs, as well as symmetrically elicited abdominal reflexes. There were no upper motor neuron signs.

Radiographs of her spine during initial assessment in our clinic revealed a right thoracic scoliosis extending from T6 to L1 and measuring 90° and a left upper thoracic scoliosis extending from T1 to T6 and measuring 51°. Thoracic kyphosis was within normal limits but lumbar lordosis was increased with an overall negative sagittal balance of her spine. The radiological evaluation excluded the presence of congenital anomalies affecting her vertebral column and chest wall. There were no features suggestive of congenital spinal stenosis and the interpedicular distance was within normal limits across all spinal segments. Her Risser grade was 2 with closed triradiate cartilage bilaterally, indicating that she had been through the most rapid stages of skeletal growth.

Due to the severity of her scoliosis the decision was made to proceed with surgical correction. In the presence of the underlying syndromic condition, a preoperative assessment was organised and this included spinal magnetic resonance imaging (MRI), and cardiac, anaesthetic and respiratory reviews.

**Figure 1:**
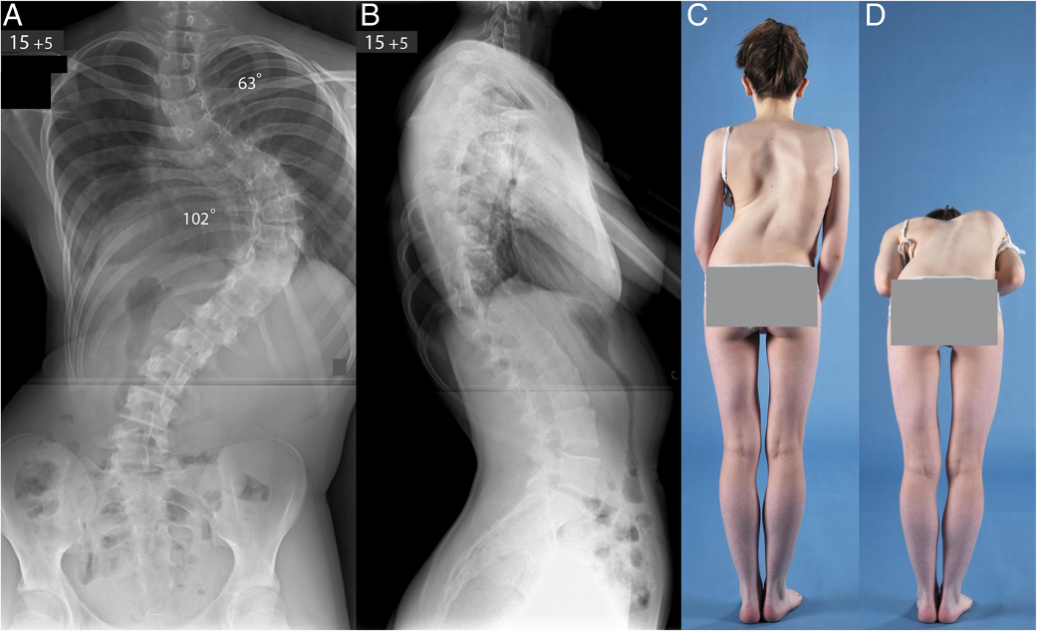
**Preoperative posteroanterior (A) and lateral (B) radiographs of the spine and clinical photographs (C, D) show a very severe double thoracic scoliosis producing thoracic translocation and spinal decompensation to the right with a marked ipsilateral rib hump and waistline asymmetry.**

Her spinal MRI demonstrated no intraspinal anomalies, normal appearance of the pedicles and no evidence of spinal stenosis. The cardiology evaluation including electrocardiogram and cardiac ultrasound showed normal function. The respiratory review including chest radiographs, capillary blood gas sample and sleep studies demonstrated a restrictive pulmonary disease with forced expiratory volume in 1 second 51% and forced vital capacity 62% predicted. The anaesthetic evaluation did not demonstrate any significant airway anomaly that could complicate intubation and confirmed the patient’s fitness to undergo scoliosis surgery. Blood test results including full blood count, urea, electrolytes, liver function tests, C-reactive protein, and coagulation screen were within normal limits. At the time of surgery, 5 months following her initial clinical presentation, progression of both thoracic curves was noticed to 102° and 63° (Figure 1). Both curvatures were significantly rigid as evidenced by supine maximum traction radiographs.

Our patient underwent a combined single-stage anterior and posterior spinal arthrodesis extending from T2 to L4 vertebrae with the use of pedicle hook, sublaminar wires and pedicle screws, and rod instrumentation at age 15 years and 5 months (Figure 1). The anterior procedure included an open right thoracotomy through an incision along the length of the 8th rib which was excised subperiosteally. Her spine was exposed from T5 to T12 and 7-level discectomies with anterior thoracoplasties that were performed to increase flexibility and allow correction of the rigid deformity. During the posterior stage, a subperiosteal exposure of her spine to the tips of the transverse processes with extensive facetectomies was performed in order to further mobilise the curve. This was followed by an interfacetal and intertransverse arthrodesis using locally harvested bone from the spinous processes and supplemented by autologous rib graft. Posterior instrumentation was used to correct the deformity and the corrective manoeuvers included apical segmental translation, rod de-rotation, as well as proximal/distal distraction/compression of the construct. Intraoperative spinal cord monitoring was performed throughout the surgery recording cortical and cervical somatosensory and transcranial electrical motor evoked potentials and there were no problems. Postoperatively, she was transferred to our intensive care unit (ICU) intubated with a right chest drain.

**Figure 2:**
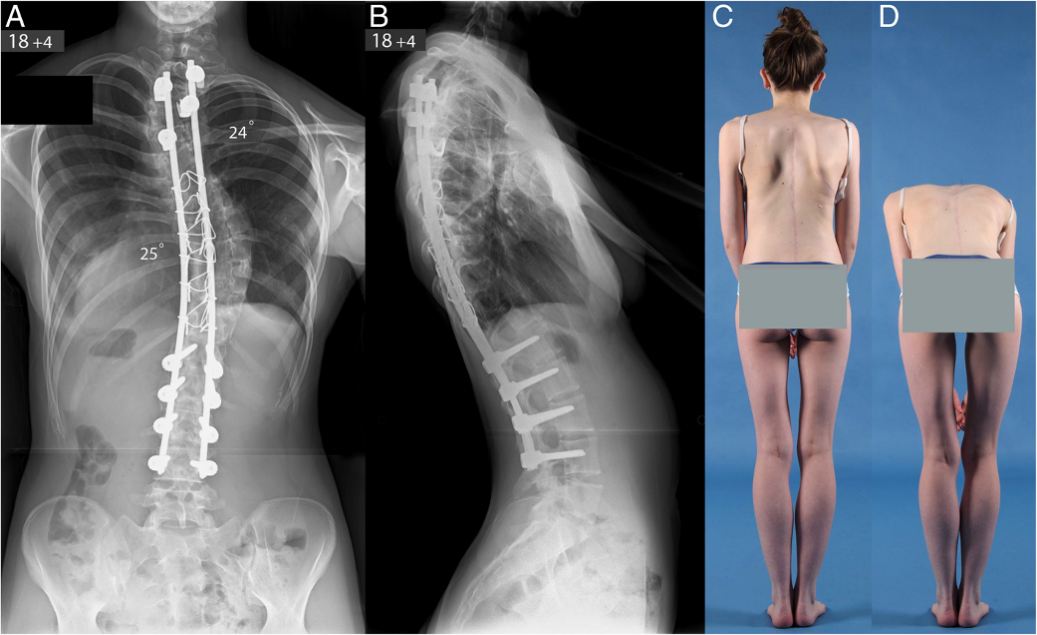
**Posteroanterior (A) and lateral (B) radiographs of the spine and clinical photographs (C, D) at latest follow-up show excellent scoliosis correction across both thoracic curves and an overall balanced spine in the coronal and sagittal planes.**

Our patient was extubated on the first postoperative day and remained in the ICU for a total of 6 days. During the initial postoperative period, her nutrition was maintained with nasogastric feedings. There were no neurological abnormalities and she gradually mobilised out of bed without external support to the spine. She received intensive respiratory physiotherapy as there was evidence of a small bilateral pleural effusion which required placement of a pigtail catheter in her left chest cavity. Non-invasive ventilation was not required. She was discharged on oral feedings 14 days after admission and made an uneventful recovery. Postoperative radiographs showed correction of her right thoracic scoliosis from 102° to 25° and the upper thoracic scoliosis from 63° to 24° with a balanced spine in the coronal and sagittal planes (Figure 2).

At latest follow-up 3 years after surgery, she had no complaints of her back and she had returned to normal activities including sports. She was skeletally mature as determined by the complete development of the iliac apophysis (Risser grade 5). Repeat spinal radiographs demonstrated no loss of scoliosis correction across the instrumented levels and no detected pseudarthrosis. There was also no evidence of add-on junctional deformity either above or below the levels of the spinal fusion.

## Discussion

TCS is a rare genetic disorder of craniofacial development that affects structures of the first and second pharyngeal arches [1–3]. The condition was described in 1889 by George Andreas Berry as a congenital neonatal deformity characterised by colobomata of the lower eyelids [4]. Edward Treacher Collins, an ophthalmologist, published in 1900 one of the earliest case reports describing ocular and peri-orbital sequelae in two patients [5]. Adolphe Franceschetti and David Klein in 1949 further defined the disorder and used the term mandibulofacial dysostosis [6]. Terms used today to describe the disease are TCS, Franceschetti-Klein syndrome, or mandibulofacial dysostosis [4].

The condition has a variable degree of phenotypic expression. There are three distinct phenotypes of TCS. Type 1 is caused by heterozygous mutation in the ‘treacle’ gene (*TCOF1*) on chromosome 5q32, whereas type 2 is caused by heterozygous mutation in the *POLR1D* gene on chromosome 13q12.2. Both types 1 and 2 are autosomal dominant disorders [1, 2]. Our patient had type 1 disorder. TCS type 3 is caused by compound heterozygous mutation in the *POLR1C* gene on chromosome 6 and is an autosomal recessive disorder [2]. A large number of mutations have been identified occurring spontaneously or in an inherited form. Combined analysis of the variants and clinical features has not demonstrated a clear relationship between genotype and phenotype [3, 7].

TCS can be diagnosed using prenatal screening ultrasonography in a mid-trimester foetus to get an adequate view of facial structures [3, 8, 9]. Three-dimensional ultrasound imaging can detect subtle malformations and assist diagnosis. In addition, amniocentesis helps differential diagnosis and can identify the underlying gene mutation [3]. Genetic testing should be suggested for high-risk families with or without ultrasound findings.

Clinical characteristics of facial dysmorphism are well described in TCS and have a bilateral distribution, even though anomalies affecting the skeletal and soft tissue structures are rarely symmetrical [3, 10]. Typical features include anti-mongoloid slant of the eyes, coloboma of the lower eyelids with a paucity of eyelashes medial to the defect, hypoplasia of the facial bones, cleft palate, malformation of the external ears, atresia of the external auditory canals, and bilateral conductive hearing loss [1, 2]. Patients have no associated developmental delay or neurological compromise [3]. Hansen *et al.* [11] observed extreme expression of TCS in a male baby with arhinia, anotia, absent zygomatic bones, hypoplastic mandibular rami, and bilateral coloboma of iris, choroid plexus, and optic nerve. Li *et al.* [12] reported the case of a patient who had additional features including encephalocele, marked malformation of the eyes, and several extracraniofacial anomalies that involved the thyroid, thymus and heart, an accessory spleen, ectopic adrenal gland tissue, and underdeveloped external genitalia.

Extracraniofacial features of TCS described in the literature, to the best of our knowledge, do not include scoliosis and there are no previous reports of its treatment as part of the condition. Our patient developed a severe double thoracic scoliosis which was first noted at the age of 14 years and progressed significantly as the effect of rapid pubertal growth. Due to the severity of the deformity, she required major scoliosis surgery which included combined single-stage anterior and posterior deformity correction and this carries potential for major postoperative morbidity. Breathing and feeding problems often occur in patients with TCS [3] and these can further complicate spinal deformity surgery. They usually present early in life and can necessitate neonatal management. Our patient had severe constrictive lung disease and developed bilateral pleural effusions in the postoperative period which required drainage and a long stay in ICU. Cardiac anomalies have been reported in patients with TCS [12] but preoperative assessment in our patient did not reveal any heart or systemic defect. Our patient had no neurological abnormalities and the pattern of her deformity resembled adolescent idiopathic scoliosis with the response to surgical treatment and long-term postoperative course being similar. Intraoperative spinal cord monitoring was performed effectively and the recorded motor and sensory traces were stable throughout the procedure. Despite the initial challenging postoperative period, our patient returned to normal activities and at skeletal maturity she had an excellent cosmetic outcome with a balanced spine and a good functional result.

## Conclusions

Scoliosis is a previously unrecognised skeletal condition in patients with TCS. It can produce a severe deformity which resembles highly progressive adolescent idiopathic scoliosis with potential for maximum deterioration around puberty and need for major surgical treatment. Surgical correction of scoliosis is indicated to restore spinal balance, prevent severe pulmonary complications and mechanical back pain, as well as improve cosmesis. Preoperative assessment needs to exclude associated feeding, cardiac, respiratory and upper airway abnormalities that can increase surgical morbidity. An excellent surgical outcome can be achieved following scoliosis correction and maintained at follow-up which results in normal function and high patient satisfaction.

## Consent

Written informed consent was obtained from the patient (after the latest clinical follow-up when she was an adult) for publication of this case report and any accompanying images. A copy of the written consent is available for review by the Editor-in-Chief of this Journal.

## Abbreviations

ICU: Intensive care unit
MRI: Magnetic resonance imaging
TCS: Treacher Collins syndrome.
